# Cell volume restriction by mercury chloride reduces M1-like inflammatory response of bone marrow-derived macrophages

**DOI:** 10.3389/fphar.2022.1074986

**Published:** 2022-12-13

**Authors:** Yen-Chieh Chuang, Shu-Yu Wu, Yu-Chuan Huang, Chung-Kan Peng, Shih-En Tang, Kun-Lun Huang

**Affiliations:** ^1^ Graduate Institute of Life Sciences, National Defense Medical Center, Taipei, Taiwan; ^2^ Institute of Aerospace and Undersea Medicine, National Defense Medical Center, Taipei, Taiwan; ^3^ School of Pharmacy, National Defense Medical Center, Taipei, Taiwan; ^4^ Department of Research and Development, National Defense Medical Center, Taipei, Taiwan; ^5^ Division of Pulmonary and Critical Care Medicine, Department of Internal Medicine, Tri-Service General Hospital, National Defense Medical Center, Taipei, Taiwan; ^6^ Graduate Institute of Medical Sciences, National Defense Medical Center, Taipei, Taiwan

**Keywords:** aquaporin, bone marrow-derived macrophages, mercury chloride, macrophage polarization, autophagy

## Abstract

Dysregulation of macrophages in the pro-inflammatory (M1) and anti-inflammatory (M2) sub-phenotypes is a crucial element in several inflammation-related diseases and injuries. We investigated the role of aquaporin (AQP) in macrophage polarization using AQP pan-inhibitor mercury chloride (HgCl_2_). Lipopolysaccharides (LPSs) induced the expression of AQP-1 and AQP-9 which increased the cell size of bone marrow-derived macrophages. The inhibition of AQPs by HgCl_2_ abolished cell size changes and significantly suppressed M1 polarization. HgCl_2_ significantly reduced the activation of the nuclear factor kappa B (NF-κB) and p38 mitogen-activated protein kinase (MAPK) pathways and inhibited the production of IL-1β. HgCl_2_ attenuated LPS-induced activation of mitochondria and reactive oxygen species production and autophagy was promoted by HgCl_2_. The increase in the light chain three II/light chain three I ratio and the reduction in PTEN-induced kinase one expression suggests the recycling of damaged mitochondria and the restoration of mitochondrial activity by HgCl_2_. In summary, the present study demonstrates a possible mechanism of the AQP inhibitor HgCl_2_ in macrophage M1 polarization through the restriction of cell volume change, suppression of the p38 MAPK/NFκB pathway, and promotion of autophagy.

## Introduction

Macrophages are highly plastic immune cells involved in both infection defense and tissue homeostasis. The dysregulation of macrophage activation is the leading cause of inflammation-related tissue injury (Laskin et al., 2011). They are classified by their response to environmental stimuli and macrophages can be polarized into a pro-inflammatory classically activated phenotype (M1) or an anti-inflammatory alternatively activated phenotype (M2) (Aggarwal et al., 2014). Induction of M1 polarization exaggerates alveolar inflammation and tissue damage (Ying et al., 2015), whereas promotion of M2 polarization mitigates pulmonary and systemic inflammation and aids in repair (Vergadi et al., 2014; Mishra et al., 2021). Macrophage polarization is mainly regulated by cytokines and chemokines and is modulated by tissue structures and physical factors in the extracellular environment (Lomas-Neira et al., 2006). The lipopolysaccharide (LPS)-induced inflammatory response and phagocytic activity of RAW264.7 cells can be modulated by altering the cell volume in various osmotic microenvironments (Hung et al., 2018). Macrophages cultured on scaffolds with smaller pore sizes promote M1 polarization (Yin et al., 2020) and mechanical elongation of macrophages leads to M2 polarization (McWhorter et al., 2015). Research on drugs that modulate physical stress in macrophages may help to develop novel therapeutic strategies against sepsis-related tissue damage.

Aquaporins (AQPs) are water channel-forming proteins that facilitate the transport of water and small non-charged molecules across the plasma membrane (Agre et al., 2002) and mediate the mechanisms of water homeostasis and osmotic regulation (Verkman et al., 2000; King et al., 2004). AQPs are also involved in shape and volume changes of various immune cells (Moon et al., 2004) and play crucial roles in the migration and phagocytosis of inflammatory cells (Meli et al., 2018). AQP has emerged as a potential target for new drug development against inflammation-related tissue damage (da Silva and Soveral, 2021). However, whether AQPs play a role in regulating macrophage polarization remains unknown. A recent study showed that knockout ablation of AQP-1 is functionally equivalent to IL-4-induced M2 polarization (Tyteca et al., 2015). In contrast, Liu *et al.* (Liu et al., 2020a) reported that AQP-1 protects against LPS-induced kidney injury by promoting macrophage M2 polarization.

Mercury ion (Hg^2+^) is an established water channel inhibitor as it effectively occludes the water transportation pore of AQPs by covalent modification of their cysteine residue (Preston et al., 1993; Zhang et al., 1993). Despite been reported as a highly toxic metal (Rice et al., 2014), recent study has shown the therapeutic potential with relatively low dose of mercury compounds against glioma (Pires et al., 2022). In immune cells, research showed that mercury chloride (HgCl_2_) inhibits the nitric oxide production of macrophage through modulate NFκB and MAPK signaling pathway (Kim et al., 2002), and attenuate caspase-1 activity, and IL-1β secretion (Ahn et al., 2018). Therefore, in the present study, we investigated the effect of HgCl_2_ on macrophage polarization.

## Materials and methods

### Preparation and culture of bone marrow-derived macrophages

Bone marrow-derived macrophages (BMDMs) were isolated from the bone marrow of C57B/6 mice aged 10–12 weeks old. All procedures were approved by the Institutional Animal Care and Use Committee of the National Defense Medical Center, Taiwan and followed the National Institutes of Health guidelines for animal care. After euthanasia, the legs of the mice were sprayed with 70% ethanol, and the femurs and tibias were dissected, separated, and transferred to a sterile flow hood. After removing the skin and muscle tissue, the bone was sprayed with 70% ethanol and the ends of the femurs and tibias were cut with sterile scissors. The bone marrow was flushed with ice-cold PBS and filtered through a 70 μm cell strainer (BD Biosciences, CA, United States) to remove solid debris. The recovered cells were cultured in high-glucose Dulbecco’s modified Eagle’s medium (DMEM; Gibco, ThermoFisher Scientific, Waltham, MA, United States) supplemented with 10% heat-inactivated fetal bovine serum (FBS; HyClone, Cytiva, Logan, UT, United States), 1% penicillin–streptomycin (Biological Industries, CT, United States), and 20 ng/ml recombinant monocytic colony-stimulating factor (M-CSF; Peprotech, Rocky Hill, NJ, United States) for 7 days to fully differentiate into macrophages.

### Experimental protocols

BMDMs were seeded into 6-well culture plates at a density of 1 × 10^6^ cells/well and subjected to the respective experiments. BMDMs were treated with 1 μg/ml LPS (*Escherichia coli* serotype 026:B6, Sigma-Aldrich, St Louis, MO, United States) or 20 μg/ml IL-4 (Peprotech) to induce macrophage polarization. To modulate cell size, BMDMs were pretreated with 20 mM NaCl (final osmolarity of 400 mOsmol) for 30 min before LPS or IL-4 stimulation. In some experiments, the cells were pretreated with 1 μM HgCl_2_ (Sigma-Aldrich). HgCl_2_ was dissolved in PBS buffer. M1/M2 polarization, expression of AQPs, cell size, production of pro-inflammatory mediators, signaling pathways, cell autophagy, and mitochondrial stress were analyzed at 8 and 24 h.

### Flow cytometry

Cells were scraped and stained with the following antibodies: APC-eFluor 780 anti-mouse F4/80 (clone BM8, eBioscience, San Diego, CA, United States), Alexa Fluor 488 anti-mouse CD11b (clone M1/70, BD Biosciences), PE anti-mouse iNOS (clone CXNFT, eBioscience), and eFluor450 anti-mouse CD38 (clone 90, eBioscience). The isotype controls were rat IgG2bκ (eBR2a) and rat IgG2bκ (DA/HA) from BD Biosciences (San Jose, CA, United States). The cells were washed and stained with Flow Cytometry Staining Buffer (eBioscience) for 30 min at 4°C. For intracellular staining, cells were fixed in IC Fixation Buffer (eBioscience) for 30 min at 4°C, permeabilized, and stained in permeabilization buffer (eBioscience). Results were obtained using the FACSVerse (BD Biosciences) and analyzed using FlowJo software (Tree Star, Ashland, OR, United States). The F4/80^+^ and CD11b^+^ cells were gated as fully differentiated macrophages.

### Quantitative real-time PCR

Total RNA was isolated from cells using the commercial kit RNA Miniprep Plus (Zymo Research Corp., Irvine, CA, United States), and cDNA was prepared using 20–100 ng/μL total RNA with the High Capacity cDNA Reverse Transcription Kit (Applied Biosystems, Foster City, CA, United States) following the manufacturer’s instructions. Real-time quantitative PCR (qPCR) was performed on the cDNA using TaqMan probes for *nos2*, *tnf*, *ccl17* (Applied Biosystems). qPCR was performed on a QuantStudio™ five System (Applied Biosystems) using the TaqMan^®^ universal Master Mix II (Applied Biosystems). Fold changes in expression were calculated by the 2^−ΔΔCt^ method using mouse gapdh (glyceraldehyde-3-phosphate dehydrogenase) as an endogenous control for mRNA expression.

### Immunofluorescence staining

Cells were cultivated in a 3.5 cm glass bottom confocal dish (SPL Life Sciences, Pocheon, Korea) at a density of 1.2 × 10^6^ cells per dish. After incubating for 24 h, the cells were fixed with 4% paraformaldehyde for 20 min and blocked with 10% bovine serum albumin (BSA) for 20 min. The cells were incubated with conjugated antibodies eFluor 660 anti-mouse F4/80 (, clone BM8, eBioscience) (1:100), PE anti-mouse iNOS (clone CXNFT, eBioscience) (1:300), and DAPI (4′,6-diamidino-2-phenylindole, Invitrogen, Carlsbad, CA, United States) for 1 h. Images were captured using a laser scanning confocal microscope (LSM 880, Carl Zeiss, United States) with a ×40 objective (original magnification).

### Western blotting assay

Cells in 6-well culture plates were lysed in a radioimmunoprecipitation assay (RIPA) cell lysis buffer containing protease and phosphatase inhibitor cocktail (Halt, ThermoFisher Scientific). To collect the nuclear fraction, cells were seeded and cultivated in 10 cm culture dishes and isolated with Nuclear and Cytoplasmic Extraction Reagent kits (NE-PER, ThermoFisher Scientific) following the manufacturer’s instructions. Protein concentrations were measured using a bicinchoninic acid protein assay kit (Pierce, ThermoFisher Scientific). The lysate (30 μg/lane) was resolved on a 10% SDS-polyacrylamide gel using a Hoefer electrophoresis blotting system (Hoefer, United States) and transferred to a 0.45 μm polyvinylidene fluoride membrane (Merck Millipore, United States). The membrane was probed with antibodies against JNK, p-JNK (Thr183/Tyr185), p38, p-p38 (Thr180/Tyr182), NF-κB (p65), p-NF-κB (p65) (Ser536), cleaved Caspase-3, LC3A/B, P62 (SQSTM1) purchased from Cell Signaling Technology (Danvers, MA, United States), PINK1 from Abcam, AQP1, and AQP9 from Biorbyt (Berkeley, CA, United States) (1:1000 dilution). β-actin (Sigma-Aldrich) and PCNA (OriGene Technologies, Rockville, MD, United States) were incubated at 1:10000 dilutions and used as internal controls. Densitometry was analyzed semi-quantitatively using ImageJ software (National Institutes of Health, Bethesda, MD, United States).

### Cytokine assays

The culture medium was collected from the 6-well culture plates. The concentration of IL-1β was quantified using ELISA kits (R&D Systems, Minneapolis, MN, United States) following the manufacturer’s instructions.

### Adherence assay

LA-4 murine lung epithelium (CCL-196) purchased from American Type Culture Collection was seeded into 24-well culture plates at 80–90% confluency. Epithelial cells were then treated with 1 μg/ml LPS for 24 h. LPS- or HgCl_2_-treated BMDMs labeled with green, fluorescent dye (Invitrogen) were seeded on top of the activated LA-4 monolayer and incubated for 1 h. Non-adherent cells were washed off with warm PBS. The remaining adhered BMDMs were counted from images captured at ×50 magnification using a fluorescence microscope (Leica DM 2500, Wetzlar, Germany). The experiment was repeated at least three times.

### Real time cell metabolism assay (seahorse assay)

Mitochondrial stress was assessed using a Seahorse XFp Extracellular Flux Analyzer (Agilent Technologies, Santa Clara, CA, United States) following the manufacturer’s instructions. BMDMs were seeded in XFp cell culture miniplates (Agilent Technologies) at a density of 3.2 × 10^5^ cells/well. After 8 h of incubation, the medium was replaced with the XF medium. Oligomycin (1 µM), carbonyl cyanide 4-(trifluoromethoxy) phenylhydrazone (FCCP, 4 µM), and antimycin A (0.5 µM) were added to the wells through a Seahorse cartridge. Real-time readings of the oxygen consumption rate (OCR) were calculated and recorded using Seahorse XFp software.

### Mitochondrial superoxide measurement

Mitochondrial reactive oxygen species (ROS) levels were measured using MitoSox-Red (Molecular Probes, Invitrogen). BMDMs were seeded in 12-well culture plates at a density of 4 × 10^5^ cells/well. The cells were double-stained with 5 μM MitoSox-Red and 50 nM MitoTracker Green FM (Molecular Probes) in HBSS for 30 min. Images were captured at ×200 and ×400 magnifications using a fluorescence microscope (Leica). The percentage of MitoSOX-positive cells was calculated at magnification of ×200.

### Statistical analysis

All results are expressed as mean ± standard error of the mean (SEM). One-way analysis of covariance (ANOVA) and Student’s t-test were used to compare differences between the study groups. Tukey’s correction was used for post-hoc comparisons. Statistical significance was set at *p* < 0.05.

## Results

### Modulation of LPS-induced M1 polarization by tonicity of medium

**FIGURE 1:**
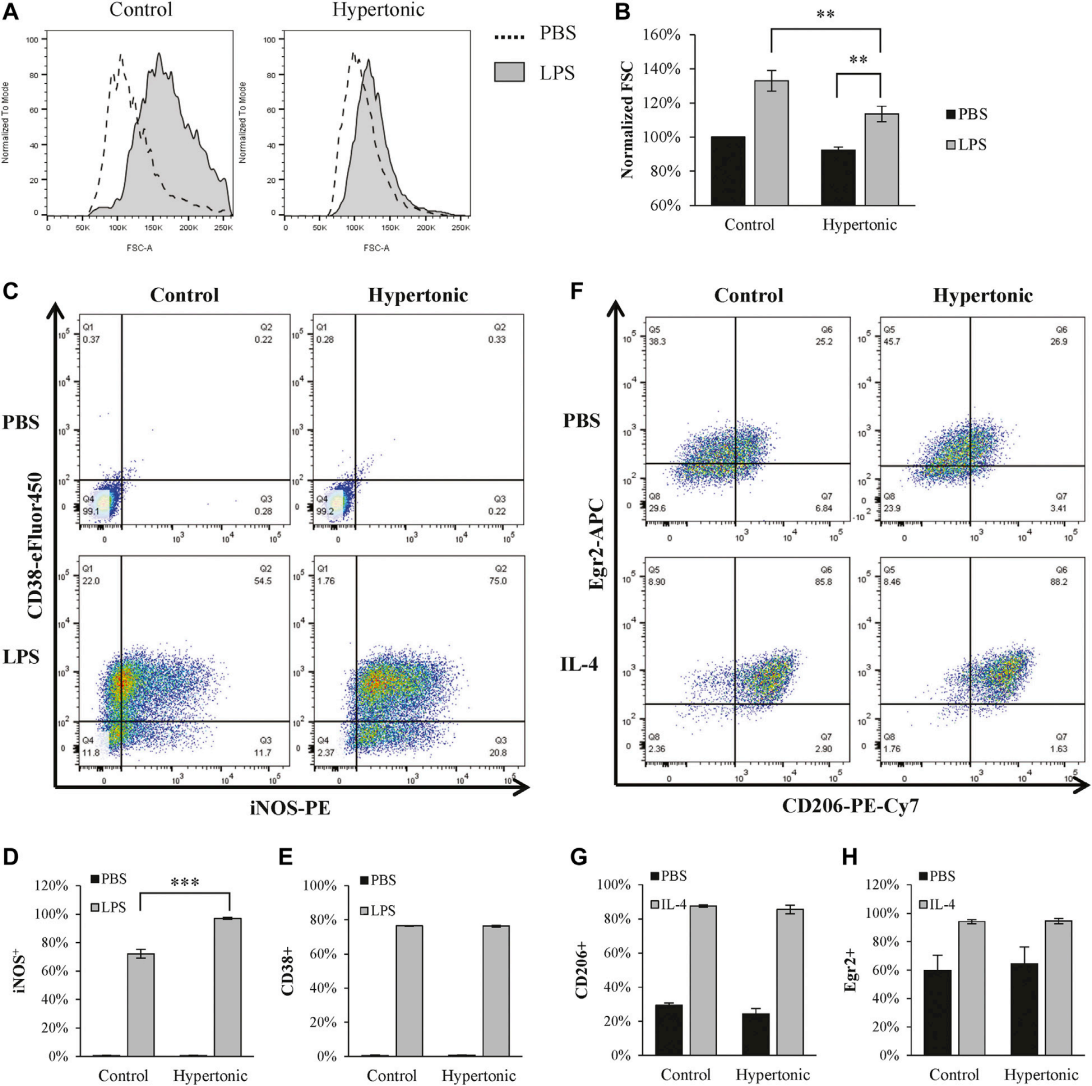
Hypertonicity induces promotes M1 polarization. **(A,B)** Normalized forward scatter (FCS) in flow cytometry was used to determine the relative cell size 24 h after tonicity (+20 mM NaCl) and LPS (1 μg/ml) treatment. (*n* = 5–7). **(C–E)** M1 polarization of BMDMs (F4/80+ and CD11b+) were estimated by iNOS and CD38 in flow cytometry (*n* = 3). **(F–H)** M2 polarization of BMDMs were estimated by CD206 and Egr2 in flow cytometry 24 h after tonicity (+20 mM NaCl) and IL-4 (20 ng/ml) treatment (*n* = 3). Values are mean ± SEM. **p < 0.01 vs. control. ***p < 0.001 vs. control.

LPS is commonly used to polarize macrophages into the M1 phenotype. LPS (1 μg/ml) significantly promoted M1 polarization in BMDM, as characterized by the increase of iNOS^+^/CD38^+^ population (Figures 1C–E). Although hypertonicity (+20 mM NaCl) restricted cell swelling (Figures 1A,B), it further enhanced LPS-induced M1 polarization (Figures 1C–E). In contrast, hypertonicity did not affect M2 polarization in BMDM (Figures 1F–H).

### Modulation of LPS-induced M1 polarization by AQP inhibition

**FIGURE 2:**
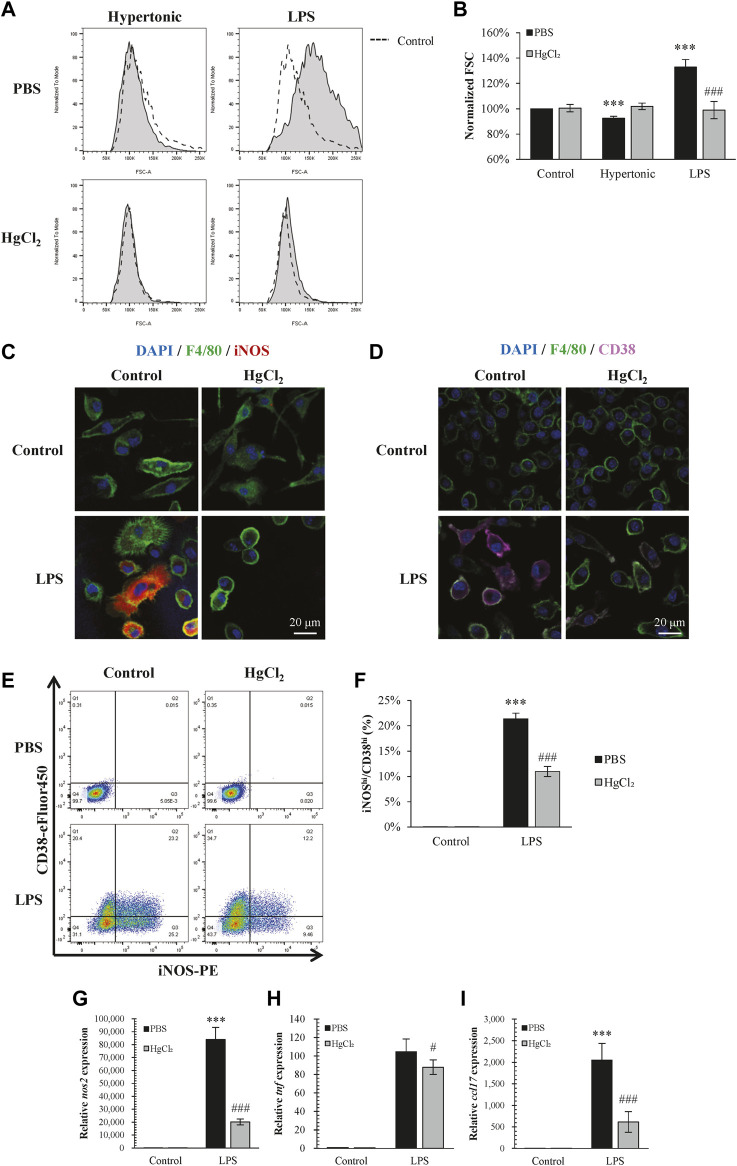
HgCl_2_ restricts lipopolysaccharides (LPS)-induced cell swelling and inhibits M1 polarization. **(A,B)** Normalized forward scatter (FCS) in flow cytometry was used to determine the relative cell size 24 h after tonicity (+20 mM NaCl) and LPS (1 μg/ml) treatment and HgCl_2_ (1 μM) pre-treatment. (*n* = 5–7). **(C,D)** Immunofluorescence image showing iNOS and CD38 staining 24 h after LPS (1 μg/ml) treatment and HgCl_2_ (1 μM) pre-treatment. **(E,F)** M1 polarization of bone marrow-derived macrophages (F4/80+ and CD11b+) were estimated by iNOS and CD38 in flow cytometry (*n* = 4–7). **(G–I)** M1-related gene expression were evaluated by TaqMan ™ qPCR assay 4 h after LPS treatment (*n* = 3). Values are mean ± SEM. ***p < 0.001 vs. control. #*p* < 0.05 vs. LPS. ###*p* < 0.001 vs. LPS.

Inhibition of AQPs by HgCl_2_ (1 μM) restricted the increase in BMDM cell size caused by LPS and also abolished cell size changes caused by hypertonicity (Figures 2A,B). On the effects to polarization, HgCl_2_ significantly inhibited LPS-induced M1 polarization (Figures 2E–I). Immunofluorescence staining showed that HgCl_2_ significantly inhibited iNOS and CD38 expression in BMDM (Figures 2C,D), the percentage of macrophage presenting high amount of iNOS (iNOS^hi^) and CD38 (CD38^hi^) signals were reduced almost by half with HgCl_2_ (Figures 2E,F). The effect of HgCl_2_ on M1 polarization was also assessed by qPCR, which showed that the expression of M1-related genes *nos2*, *tnf*, and *ccl17* was reduced by 76%, 21%, and 72%, respectively (Figures 2G–I).

### Suppression of inflammatory pathway and adherence function by AQP inhibitor

**FIGURE 3:**
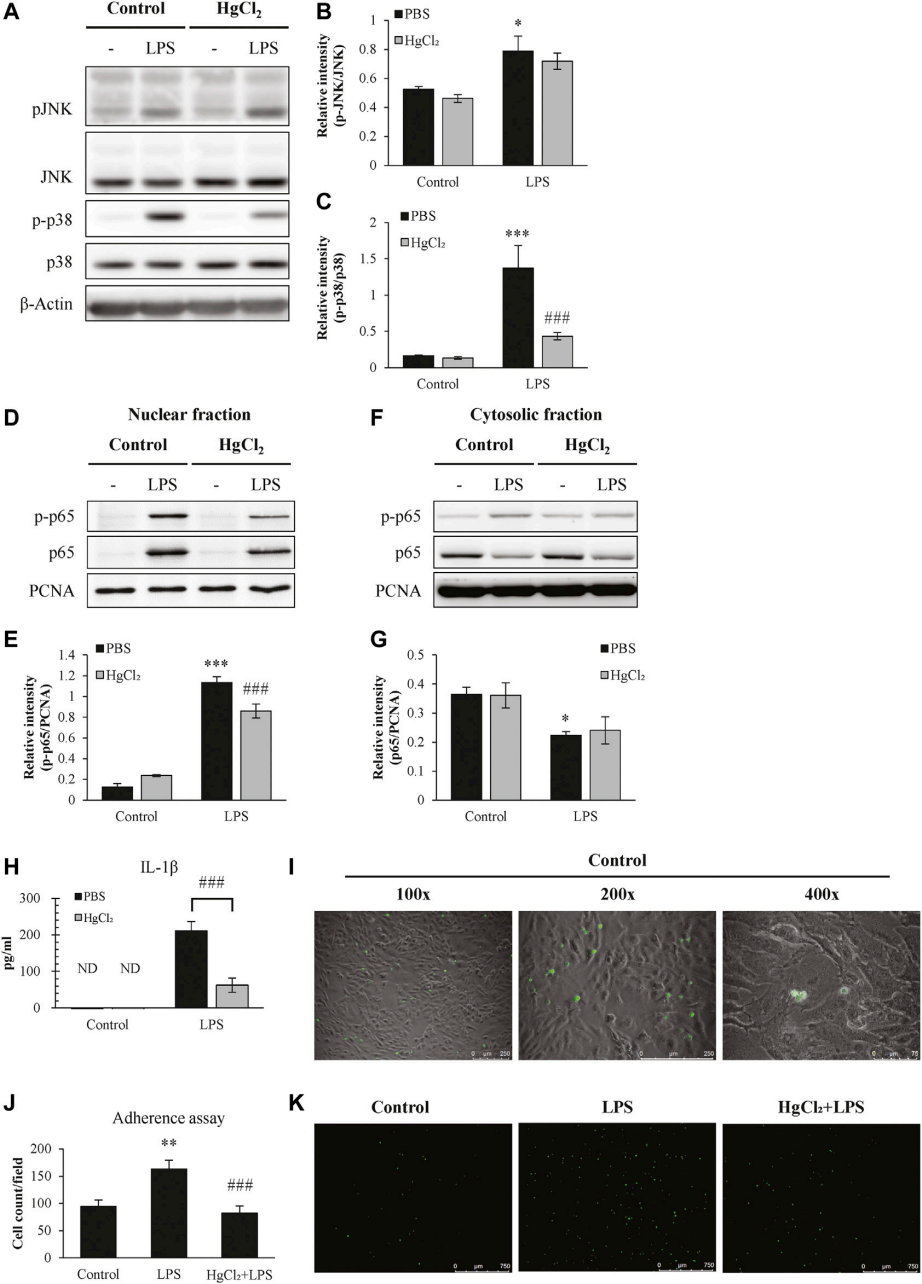
HgCl_2_ suppress inflammatory pathway and adherence function. **(A,D,F)** Inflammatory cascade 30 min after lipopolysaccharides (LPS) (1 μg/ml) and HgCl_2_ (1 μM) treatment was determined by western blot analysis. and **(B–G)** interpreted by semi quantitative densitometric analysis (n = 3–4). **(H)** The level of IL-1β production 24 h after LPS (1 μg/ml) and HgCl_2_ (1 μM) treatment. **(I)** Images of adhered BMDMs (green) on LA-4 murine lung epithelium (DIC) in different magnification (×100, 200×, and 400×). **(J,K)** Adhered BMDMs (green) in adherence assay were imaged and counted by fluorescence microscope (50×) and imageJ (n = 7–11). Values are mean ± SEM. **p = 0.001 vs. control. ***p < 0.001 vs. control. ###*p* < 0.001 vs. LPS.

Activation of nuclear factor kappa B (NF-κB) pathway and increased adhesion ability are two notably features for M1 macrophage. LPS increased the cytoplasmic abundance of phosphorylated p38 (Figures 3A–C) and the nuclear translocation of p-p65 NF-κB (Figures 3D–G) in BMDM, and produced large amounts of IL-1β (Figure 3H). LPS also significantly increased the adhesion ability of BMDM (Figures 3I–K). HgCl_2_ effectively suppressed the activation of the NF-κB and p38 MAPK pathways and inhibited the production of IL-1β (Figures 3A–H). The presence of HgCl_2_ also significantly reduced the adhesion ability (Figures 3I–K).

### HgCl_2_ reduces mitochondrial stress and ROS production in M1 BMDM

**FIGURE 4:**
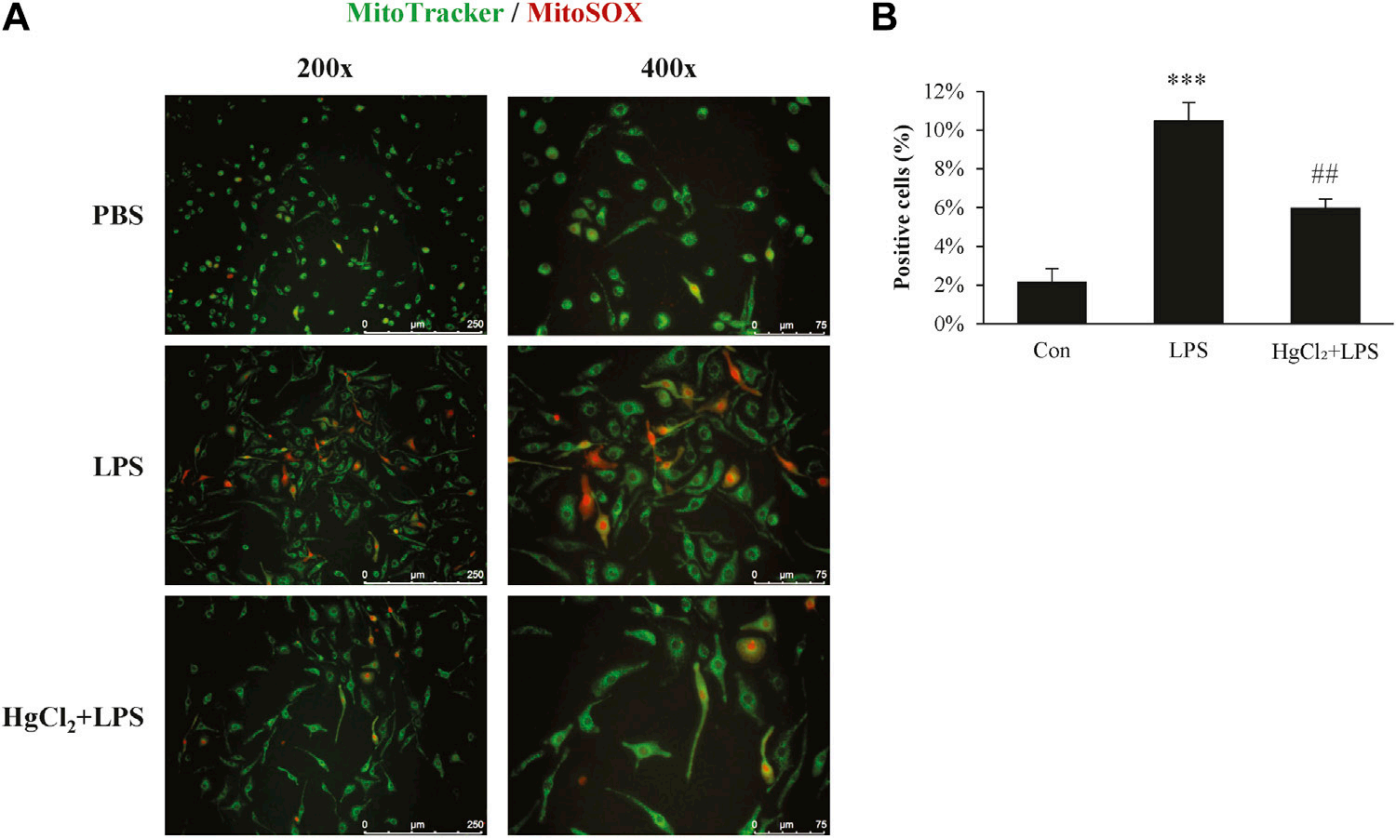
HgCl_2_ reduces mitochondrial reactive oxygen species production. **(A)** Immunofluorescence image of MitoSOX staining 8 h after lipopolysaccharides (LPS) (1 μg/ml) and HgCl_2_ (1 μM) treatment. **(B)** The percentage of MitoSOX positive cells per field in ×200 magnification was analyzed with imageJ (*n* = 7–11). Values are mean ± SEM. ***p < 0.001 vs. control. ##*p* < 0.01 vs. LPS.

Mitochondrial ROS production plays an important role in macrophage immunity; however, over-activated mitochondria are associated with cellular stress and dysfunction. Mitochondrial ROS production was estimated using MitoSOX staining. LPS induced substantial mitochondrial ROS production in BMDM (Figure 4A) and significantly increased the percentage of MitoSOX-positive cells (Figure 4B). The LPS-induced mitochondrial ROS production was significantly suppressed by HgCl_2_ treatment (Figure 4).

**FIGURE 5:**
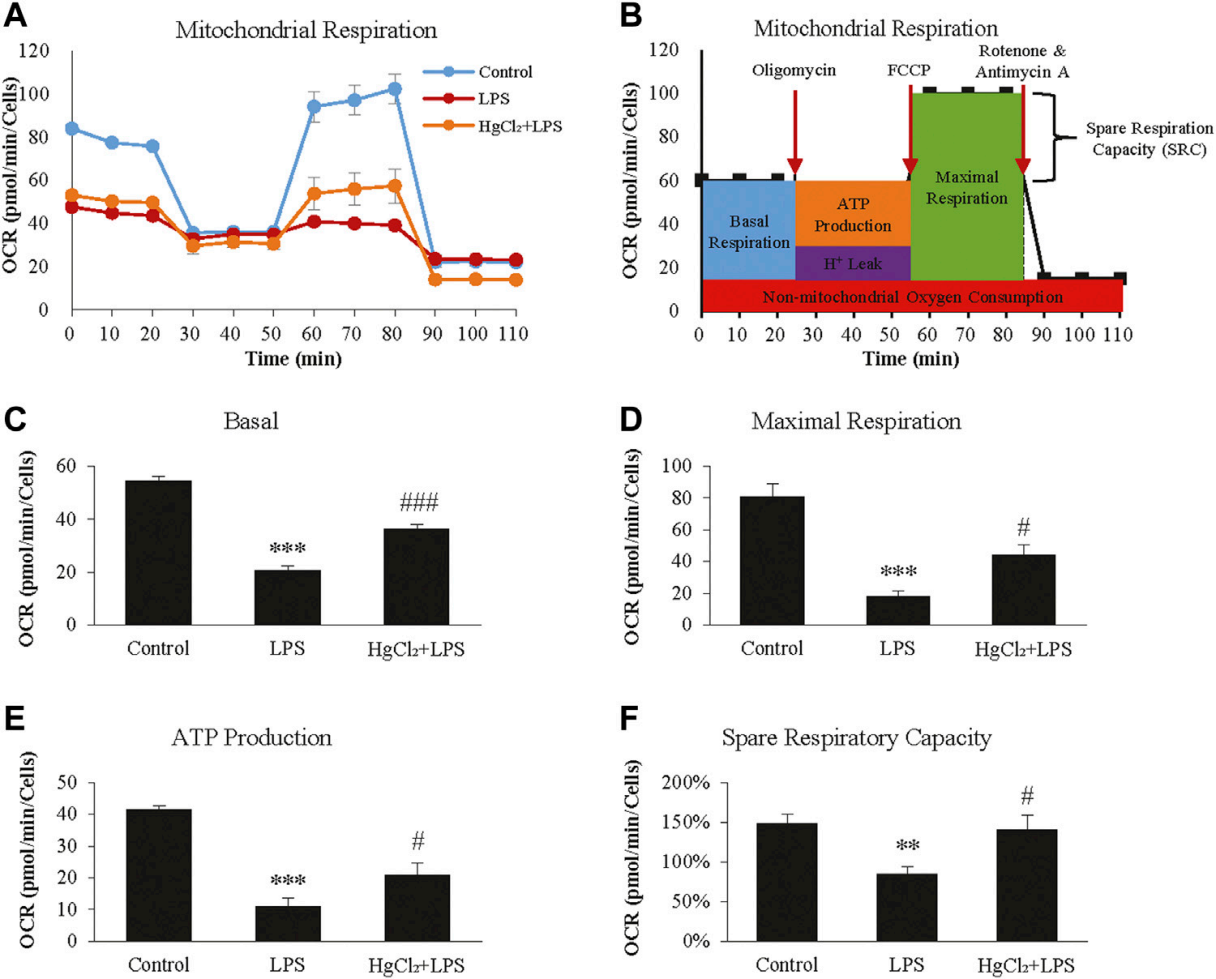
HgCl_2_ reduces lipopolysaccharides (LPS)-induced mitochondrial stress of bone marrow-derived macrophages (BMDM). **(A)** Oxygen consumption rates (OCR, in pmol/min) profile of BMDMs after 8 h of LPS (1 μg/ml) and HgCl_2_ (1 μM) treatment. **(B)** Schematic diagram of how the fundamental parameters of mitochondrial function measured by XF Cell Mito Stress Test Kit. Calculated basal **(C)**, maximal respiration **(D)**, ATP production **(E)**, and spare respiratory capacity **(F)**. All measurements were normalized for cell count (n = 4). Values are mean ± SEM. **p < 0.01 vs. control. ***p < 0.001 vs. control. #*p* < 0.05 vs. LPS. ###*p* < 0.001 vs. LPS.

The Seahorse XF Cell Mito Stress Test was used to calculate the fundamental parameters of mitochondrial function (Figure 5A). LPS significantly suppressed oxygen consumption in BMDM under basal conditions (Figure 5C) and during maximal respiration (Figure 5D). LPS also reduced the ATP-linked OCR and spare respiratory capacity (Figures 5E,F). HgCl_2_ significantly restored mitochondrial function (Figures 5C–F).

### HgCl_2_ upregulates the autophagy in M1 BMDMs

**FIGURE 6:**
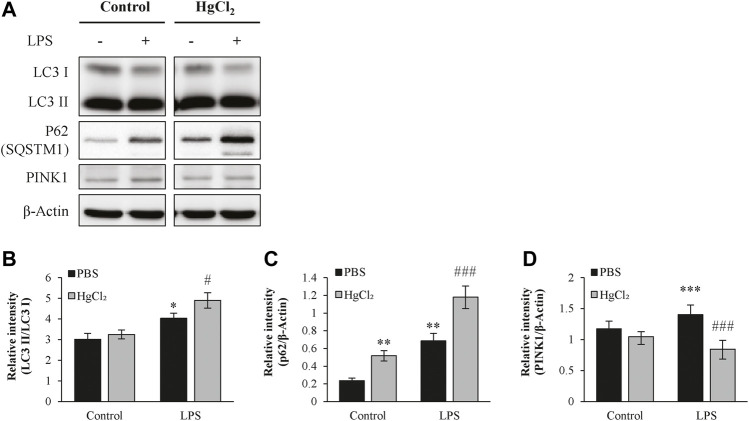
HgCl_2_ upregulates the autophagy in M1 bone marrow-derived macrophages (BMDMs). **(A)** Autophagy of BMDMs 8 h after LPS (1 μg/ml) and HgCl_2_ (1 μM) treatment was determined by western blot analysis and **(B–D)** interpreted by semi quantitative densitometric analysis (*n* = 4–6). Bafilomycin A1 (100 nM) were cotreated with LPS to normalize autophagy flux. Values are mean ± SEM. *p < 0.05 vs. control. **p < 0.01 vs. control. ***p < 0.001 vs. control. #*p* < 0.05 vs. LPS. ###*p* < 0.001 vs. LPS.

Autophagy is a recycling mechanism of cellular homeostasis responsible for maintaining healthy mitochondria. LPS increased light chain three II/light chain three I level in the presence of bafilomycin A1 (Figures 6A,B) and caused substantial p62/SQSTM1 abundance in BMDM (Figures 6A,C). HgCl_2_ further augmented the LPS-induced increase in autophagic flux (Figures 6A–C). LPS also stimulated the expression of mitophagy marker PTEN-induced kinase 1 (PINK1), indicating mitochondrial stress. Pre-treatment of BMDM with HgCl_2_ reduced LPS-induced PINK1 abundance, suggesting that the present of AQP inhibitors alleviated mitochondrial stress (Figure 6D).

## Discussion

Our previous studies in primary alveolar macrophages and RAW 264.7 cells, have found that cell size is one of the critical parameters determining the inflammatory response to LPS treatment (Hung et al., 2018; Hung et al., 2020). Shrinking the size of macrophages with osmolarity differences is associated with a lower activated IKK-NfκB cascade and less TNF-α and IL-6 secretion following LPS treatment. In contrast, further enlargement of macrophage cell size with a hypoosmotic medium enhances its inflammatory responses. However, in the present study with BMDMs, M1 polarization induced by LPS seems to be augmented by hypertonicity. This may contribute to the differences in the macrophage origin. Studies have shown that inflammasome activity is boosted with high osmolarity in BMDM (Ip and Medzhitov, 2015), but significantly suppressed in alveolar or peritoneal macrophages (Rabolli et al., 2014).

HgCl_2_ is well established as an AQP inhibitor, and mercury physically blocks water transportation pores by thiol binding to its pore-localized cysteine (Savage and Stroud, 2007; Yool et al., 2010). Although mercury is considered a highly toxic metal, studies have shown that low-dose treatment with HgCl_2_ (up to 10 μM) is not toxic to macrophages (Rabolli et al., 2014) and could even be beneficial against inflammation. In immune cells, studies have shown that the introduction of HgCl_2_ alters the inflammatory response of macrophages by modulating the NF-κB and MAPK signaling pathways (Kim et al., 2002), attenuate NLRP3 related inflammasome activation and caspase-1 and IL-1β secretion (Ahn et al., 2018). In the present study, the viability of BMDMs showed no significant reduction up to 5 μM HgCl_2_, and the real-time cell metabolism assay revealed that mitochondrial activity was restored with 1 μM HgCl_2_, further suggesting that metabolic integrity was intact.

Autophagy is a highly conserved mechanism in eukaryotic cells that maintains cell homeostasis and organelle quality and can be stimulated by nutrient starvation, genotoxic agents, cytokines, or oxidative stress. Studies have revealed that exposure to mercury promotes autophagy in hepatocytes and stem cells (Chang et al., 2013; Chatterjee et al., 2014), and in macrophages, autophagy is crucial for defense against infection (Gutierrez et al., 2004; Nakagawa et al., 2004). A decrease in autophagy activity correlates positively with the inflammatory response. A recent study found that macrophage autophagy might play an important role in macrophage differentiation and polarization. Liu *et al.* demonstrated that autophagy deficiency in macrophages promotes M1 polarization and suppresses the M2 polarization potential (Liu et al., 2015). Our results confirm this finding using the opposite approach. The initiation of autophagy is a common response of macrophages to LPS stimulation (Xu et al., 2007; Delgado et al., 2008), but further boosting autophagy activity with HgCl_2_ is associated with reduced M1 polarization.

NF-κB activity is essential for LPS-mediated polarization to the M1 phenotype and is widely used to simulate pulmonary infection in acute lung injury (ALI) models. NF-κB is central to many inflammatory macrophage functions, such as the expression and secretion of iNOS, TNF-α, IL-1, IL-6, and bacterial phagocytosis (Xie et al., 1994; Goldring et al., 1995; Sha et al., 1995). The MAPK pathways, JNK and p38, are crucial in regulating the activation of NF-κB, and the phosphorylation of JNK and p38 dissociates the bonding of cytosolic NF-κB and IκB, resulting in the nuclear translocation of NF-κB, thus promoting the correlated gene expression (Liu et al., 2020b). Moreover, both p38 and JNK are associated with the regulation of apoptosis and autophagy. However, the roles of p38 and JNK in regulating apoptosis and autophagy are still unclear, both of which play a dual role in regulating autophagy as positive and negative regulators (Sui et al., 2014). Our results demonstrated that although the translocation of NF-κB was suppressed by HgCl_2_ treatment, it reduced only the phosphorylation of p38, but not JNK. Similar results were found in a study on microglia, in which the inhibition of p38 restored the kinase activity of ULK1, an initiation complex of the autophagic cascade in microglia, thus increasing autophagy activity (He et al., 2018).

Macrophages are the frontline of innate and adaptive immune defense against pathogen infection, and regulate immune responses. However, during inflammation, macrophage mitochondria are major targets of oxidative stress. Mitochondrial damage in macrophages is associated with apoptosis, metabolic switching, and systemic inflammatory responses (Ramond et al., 2019). In ALI, the level of mitochondrial dysfunction and ROS production in macrophages correlates positively with acute lung injury outcomes (Deng et al., 2017; Dong and Yuan, 2018). Recent studies have shown that artificially transferring healthy mitochondria from mesenchymal stem cells to damaged tissue protects against mitochondria-related injuries such as ALI (Islam et al., 2012), cardiac ischemia/reperfusion injury (Han et al., 2016) and cortical neurons after stroke (Babenko et al., 2015). These findings further emphasize the importance of mitochondrial integrity in inflammatory diseases. Our results show that treatment with HgCl_2_ significantly reduces the generation of mitochondrial ROS following LPS stimulation. Similar results were found in the study by H. Ahn *et al.* in an inflammasome model. They demonstrated that the introduction of HgCl_2_ prevents mitochondrial ROS production and cytosolic release of mitochondria DNA (Ahn et al., 2018). However, our study further evaluated mitochondrial health using a real-time cell metabolism assay and showed that HgCl_2_ also restored mitochondrial function during respiration.

**FIGURE 7:**
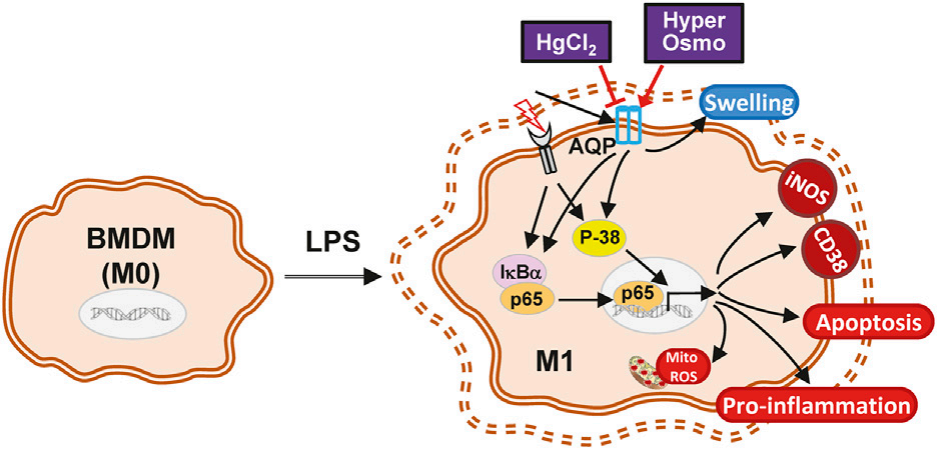
The schematic diagram of the effect of HgCl_2_ on bone marrow-derived macrophages in inflammatory response. The stimulation from LPS triggers the M1 polarization of BMDMs, activating the inflammatory cascade and causing the swelling of the cell. Although hypertonicity and HgCl_2_ induction are both able to restrict cell enlargement, the AQP inhibition effect of HgCl_2_ can significantly reduce the M1 phenotype of BMDMs. HgCl_2_ suppress the NF-κB activation mainly through the downregulation of p38/MAPK pathway, reducing the translocation of NF-κB into nucleus. Elevated autophagy levels may also help to recycle over-activated mitochondria, preventing mitochondrial ROS stress, and maintaining cell metabolic stability.

In summary, the present study demonstrates a possible mechanism of action for the AQP inhibitor HgCl_2_ in macrophage polarization. The LPS-induced M1 activation of macrophages was attenuated by the inhibitory effect of HgCl_2_. It reduces cytokine production, adhesion, apoptosis, and mitochondrial ROS production. NF-κB inhibition is mainly mediated by the downregulation of p38 MAPK signaling. The evoked autophagy level can accelerate the recycling of damaged mitochondria, regain mitochondrial activity, and prevent mitochondrial damage (Figure 7). However, additional research is needed to clarify the inhibition specificity of HgCl_2_ on the AQP family and the molecular interactions between AQPs and MAPKs to provide a more complete view of this mechanism.

## Data Availability

The original contributions presented in the study are included in the article/supplementary materials, further inquiries can be directed to the corresponding author.
