# Corrigendum: Cell volume restriction by mercury chloride reduces M1-like inflammatory response of bone marrow-derived macrophages

**DOI:** 10.3389/fphar.2023.1210999

**Published:** 2023-05-09

**Authors:** Yen-Chieh Chuang, Shu-Yu Wu, Yu-Chuan Huang, Chung-Kan Peng, Shih-En Tang, Kun-Lun Huang

**Affiliations:** ^1^ Graduate Institute of Life Sciences, National Defense Medical Center, Taipei, Taiwan; ^2^ Institute of Aerospace and Undersea Medicine, National Defense Medical Center, Taipei, Taiwan; ^3^ School of Pharmacy, National Defense Medical Center, Taipei, Taiwan; ^4^ Department of Research and Development, National Defense Medical Center, Taipei, Taiwan; ^5^ Division of Pulmonary and Critical Care Medicine, Department of Internal Medicine, Tri-Service General Hospital, National Defense Medical Center, Taipei, Taiwan; ^6^ Graduate Institute of Medical Sciences, National Defense Medical Center, Taipei, Taiwan

**Keywords:** aquaporin, bone marrow-derived macrophages, mercury chloride, macrophage polarization, autophagy

In the published article, there was an error regarding the **Affiliations** for Kun-Lun Huang. As well as having affiliations 5 and 6, they should also have “1Graduate Institute of Life Sciences, National Defense Medical Center, Taipei, Taiwan.”

The authors apologize for this error and state that this does not change the scientific conclusions of the article in any way. The original article has been updated.

